# Supplementary material for: Enhanced excitability but mature action potential waveforms at mossy fiber terminals of young, adult-born hippocampal neurons in mice
Source: Commun Biol. 2023 Mar 18;6:290. doi: 10.1038/s42003-023-04678-5 (PMC10024705; doi:10.1038/s42003-023-04678-5)
Supplement: Supplementary file 2 — Supplementary Figures [file 42003_2023_4678_MOESM2_ESM.pdf]

Supplementary Figures

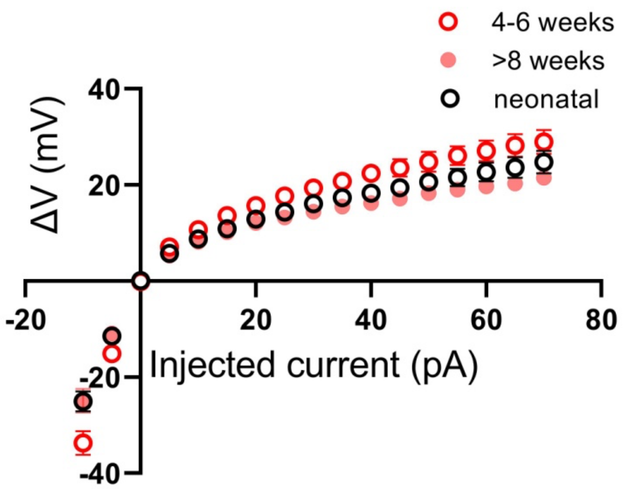

Figure S1. Related to Fig. 2. Steady-state voltage response plotted as a function of injected current amplitude for the three groups of mossy fiber terminals. Voltages were measured at the end of the 500-ms current step. Note that all groups showed marked rectification, but that the curve for boutons from immature adult-born neurons has steeper slopes consistent with higher input resistance ($Slope_{(-10–0 pA)}$ = 3.4, 2.5, and 2.5 GOhm and $Slope_{(10–70 pA)}$ = 0.30, 0.21, and 0.25 GOhm for 4-6w, 8+w, and neonatal groups, respectively; see Fig. 2C).

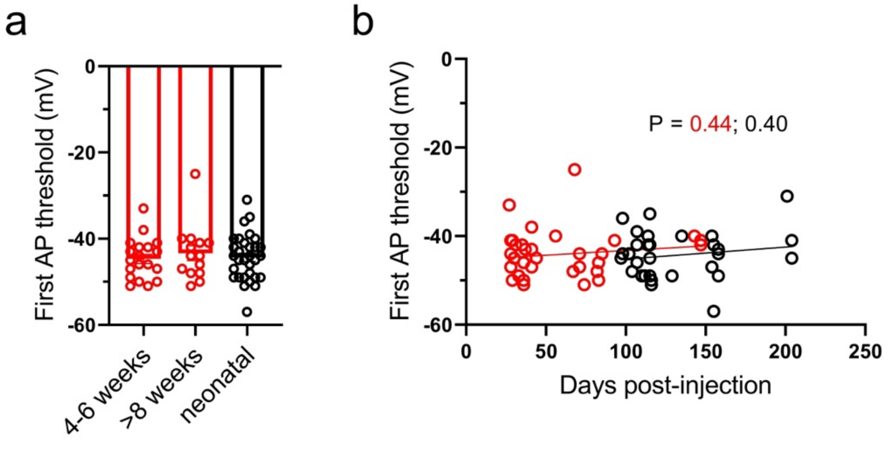

Figure S2. Related to Fig. 2. Threshold of action potentials elicited by minimum current injection do not differ between groups of boutons (experiments like in Fig. 2G). (a) AP threshold for the three groups (Kruskal Wallis, P = 0.84; 4-6w vs 8+w, P = 0.57) and (b) plotted as a function of cell age (R^2 = 0.02, P = 0.44, F(1, 32) = 0.61 for adult-born neurons). Bars reflect mean ± standard error.

Supplementary Figures

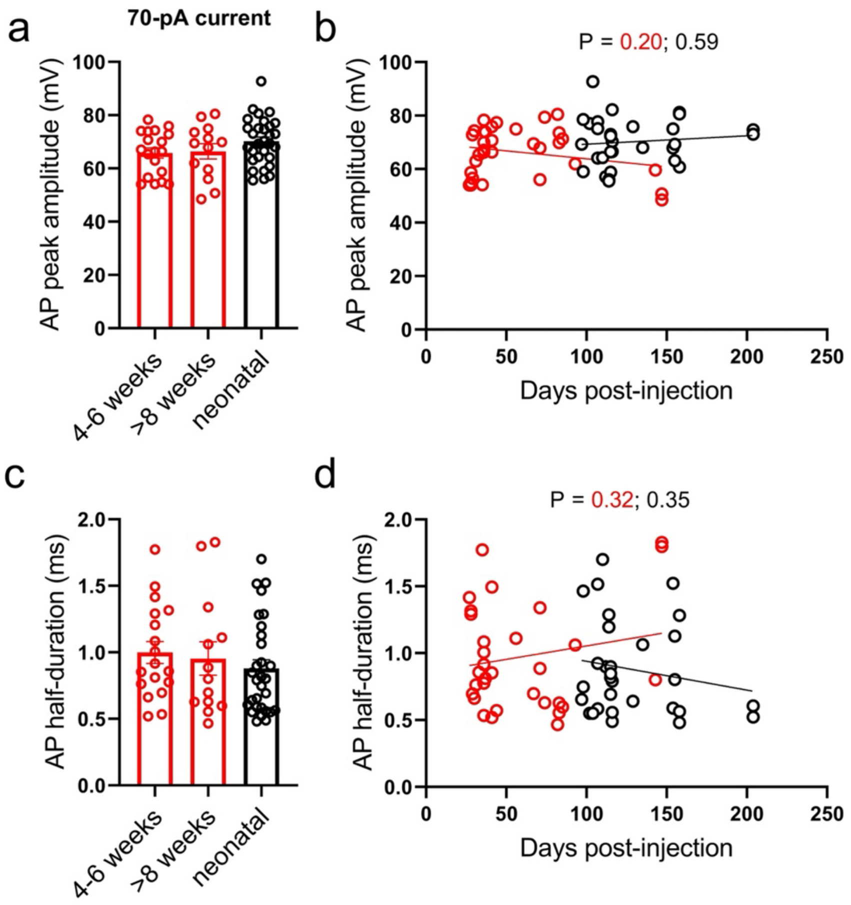

Figure S3. Related to Fig. 2. Peak amplitude and half-duration of action potentials elicited by 70 pA (500-ms) current injections (experiments like in Fig. 2G). (a-d) Average peak amplitude (a; one-way ANOVA, P = 0.20, F(2, 59) = 1.7; 4-6w vs 8+w, P = 0.85) and half-duration (c; one-way ANOVA, P = 0.55, F(2, 58) = 0.60; 4-6w vs 8+w, P = 0.74) of action potentials evoked in boutons from the three groups of cells, and plotted as a function of cell age (b,d; R^2 = 0.05, P = 0.20, F(1, 31) = 1.7 and R^2 = 0.03, P = 0.32, F(1, 30) = 1.0, respectively for adult-born neurons). Note that no consistent differences were observed between the groups. Bars reflect mean ± standard error.

Supplementary Figures

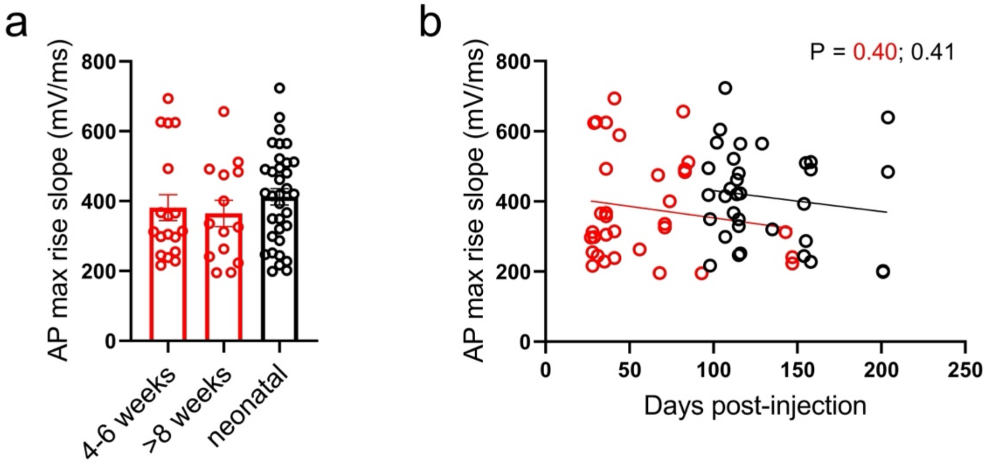

Figure S4. Related to Fig. 3. Maximal rate of rise of action potentials for boutons from the three groups of cells (a; one-way ANOVA, $P = 0.5$, $F(2, 63) = 0.64$; 4-6w vs 8+w, $P = 0.8$) and plotted as a function of cell age (b; $R^2 = 0.02$, $P = 0.4$, $F(1, 32) = 0.75$ for adult-born neurons). Bars reflect mean ± standard error.

Supplementary Figures

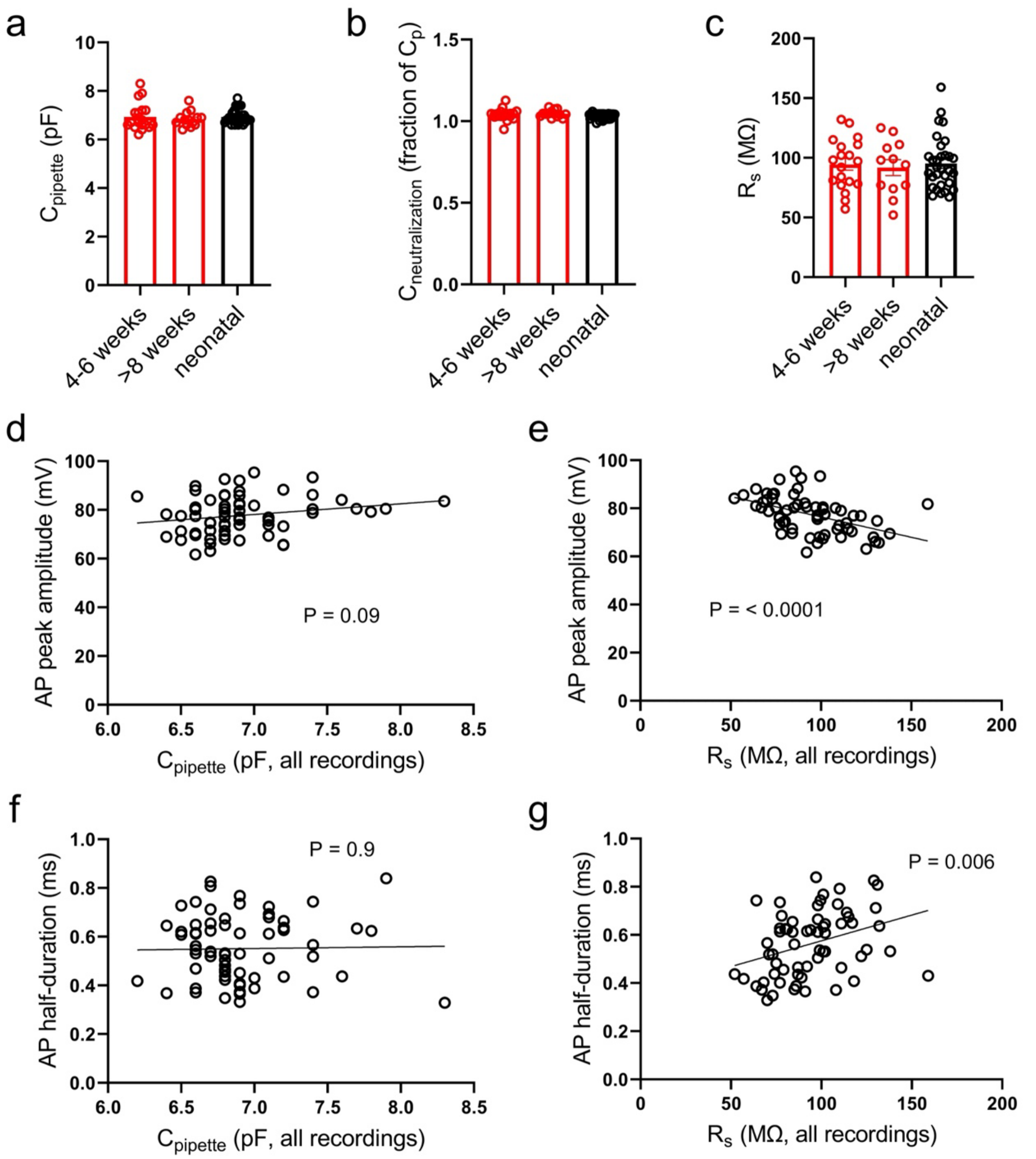

Figure S5. Related to Fig. 3. Dependence of AP waveform at mossy fiber terminals on recording resistance and pipette capacitance. (a-c) Fast pipette capacitance ($C_{pipette}$, a), fraction of $C_{pipette}$ neutralized during current-clamp experiments (b), and series resistance (R_s, c) were not different for the three groups of mossy fiber terminal recordings (Kruskal-Wallis, P = 0.4; 4-6w vs 8+w, P = 0.95 for $C_{pipette}$; one-way ANOVA, P = 0.14, F(2, 63) = 2.0; 4-6w vs 8+w, P = 0.5 for $C_{neutralization}$; Kruskal-Wallis, P = 0.9; 4-6w vs 8+w, P = 0.7 for R_s). (d-g) Neither peak amplitudes (d) nor half-durations (f) of action potentials varied as a function of pipette capacitance (R^2 = 0.04, P = 0.09, F(1, 65) = 3.0; R^2 = 0.0004, P = 0.9, F(1, 65) = 0.026, respectively), but both were significantly related to series resistance (E, R^2 = 0.22, P < 0.001, F(1, 61) = 18; g, R^2 = 0.12, P = 0.006, F(1, 61) = 8.1). For analyses in (d-g) all mossy fiber terminal recordings are pooled. Bars reflect mean ± standard error.

Supplementary Figures

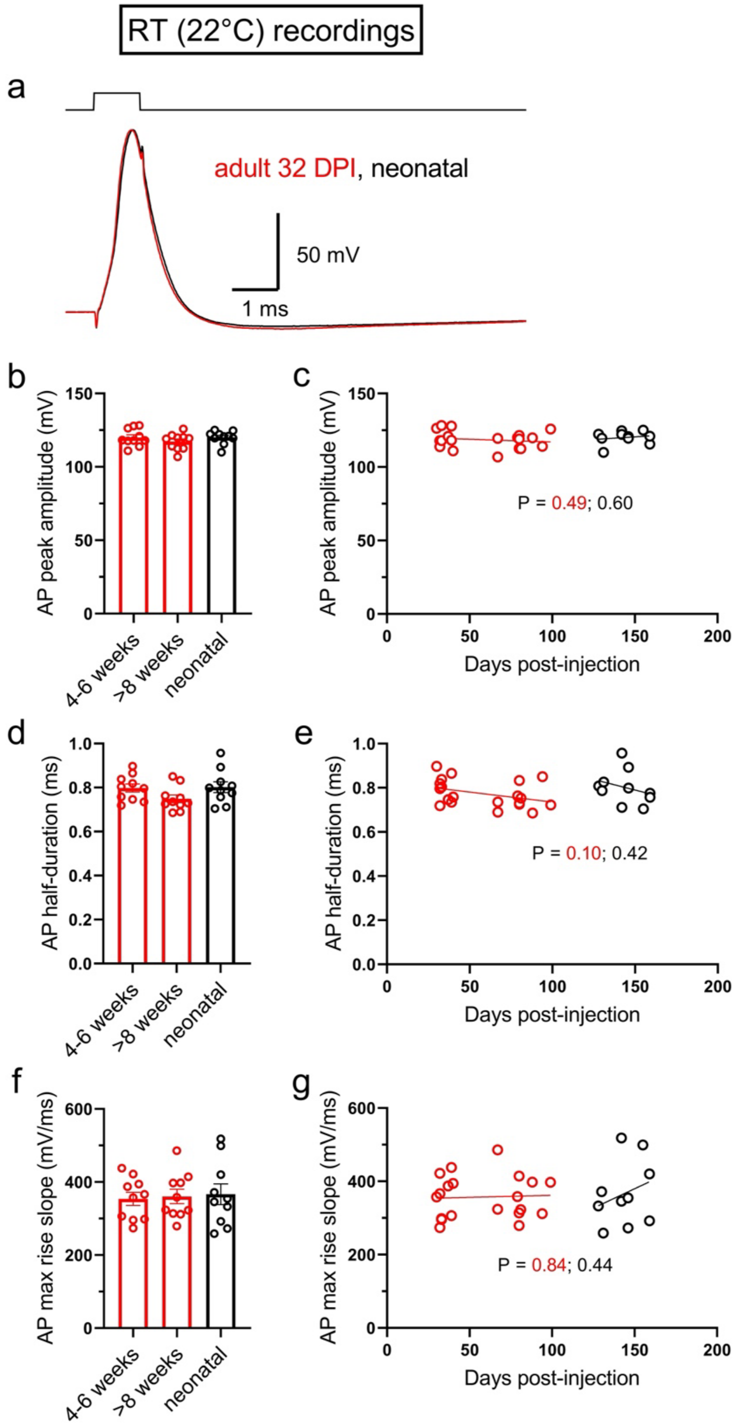

Figure S6. Related to Figure 3. Mature action potentials at boutons from young adult-born granule neurons recorded at 22 degrees C. (a) Example action potentials recorded in mossy fiber boutons from either adult- (32 DPI, red) or neonatal-born (black) granule neuron evoked by a brief current injection (300 pA, 1 ms). (b-g) Average peak amplitude (b; Kruskal Wallis, P = 0.42; 4-6w vs 8+w, P = 0.45) half-duration (d; one-way ANOVA, P = 0.14, F(2, 27) = 2.1; 4-6w vs 8+w, P = 0.10) and maximal rate of rise (f; one-way ANOVA, P = 0.92, F(2, 27) = 0.08; 4-6w vs 8+w, P = 0.83) of action potentials evoked in boutons from the three groups of cells, and

plotted as a function of cell age (c,e,g; R^2 = 0.03, P = 0.49, F(1, 18) = 0.50, R^2 = 0.14, P = 0.10, F(1, 18) = 2.9, and R^2 = 0.0023, P = 0.84, F(1, 18) = 0.04 respectively for adult-born neurons). Note that action potentials were larger at 22 than at 32°C, but that no consistent differences in waveforms were observed between the groups. Bars reflect mean ± standard error.
