# Supplementary material for: Enhanced excitability but mature action potential waveforms at mossy fiber terminals of young, adult-born hippocampal neurons in mice
Source: Commun Biol. 2023 Mar 18;6:290. doi: 10.1038/s42003-023-04678-5 (PMC10024705; doi:10.1038/s42003-023-04678-5)
Supplement: Supplementary file 3 — Description of Additional Supplementary Files [file 42003_2023_4678_MOESM3_ESM.pdf]

Description of Additional Supplementary Files

File name: Supplementary Data 1
Description: The data for all graphs and analyses.
