# Supplementary material for: Enhanced excitability but mature action potential waveforms at mossy fiber terminals of young, adult-born hippocampal neurons in mice
Source: Commun Biol. 2023 Mar 18;6:290. doi: 10.1038/s42003-023-04678-5 (PMC10024705; doi:10.1038/s42003-023-04678-5)
Supplement: Supplementary file 5 — Reporting Summary [file 42003_2023_4678_MOESM5_ESM.pdf]

nature portfolio

Corresponding author(s): Jason S Snyder

Last updated by author(s): Mar 6, 2023

Reporting Summary

Nature Portfolio wishes to improve the reproducibility of the work that we publish. This form provides structure for consistency and transparency in reporting. For further information on Nature Portfolio policies, see our Editorial Policies and the Editorial Policy Checklist.

Statistics

For all statistical analyses, confirm that the following items are present in the figure legend, table legend, main text, or Methods section.

n/a	Confirmed	
☐	☒	The exact sample size (*n*) for each experimental group/condition, given as a discrete number and unit of measurement
☐	☒	A statement on whether measurements were taken from distinct samples or whether the same sample was measured repeatedly
☐	☒	The statistical test(s) used AND whether they are one- or two-sided<br>*Only common tests should be described solely by name; describe more complex techniques in the Methods section.*
☒	☐	A description of all covariates tested
☐	☒	A description of any assumptions or corrections, such as tests of normality and adjustment for multiple comparisons
☐	☒	A full description of the statistical parameters including central tendency (e.g. means) or other basic estimates (e.g. regression coefficient) AND variation (e.g. standard deviation) or associated estimates of uncertainty (e.g. confidence intervals)
☐	☒	For null hypothesis testing, the test statistic (e.g. *F*, *t*, *r*) with confidence intervals, effect sizes, degrees of freedom and *P* value noted<br>*Give P values as exact values whenever suitable.*
☒	☐	For Bayesian analysis, information on the choice of priors and Markov chain Monte Carlo settings
☒	☐	For hierarchical and complex designs, identification of the appropriate level for tests and full reporting of outcomes
☐	☒	Estimates of effect sizes (e.g. Cohen's *d*, Pearson's *r*), indicating how they were calculated

Our web collection on statistics for biologists contains articles on many of the points above.

Software and code

Policy information about availability of computer code

Data collection	Data were collected using Multiclamp 700B amplifier (Axon Instruments) run by Clampex 10.7 software (PClamp, Molecular Devices) and digitized using Digidata 1550B analog-digital converter (Axon Instruments).
Data analysis	Data were analyzed using Clampfit 10.7 (Molecular Devices) Igor Pro (Wavemetrics), and Stimfit software, tabulated in Microsoft Excel, and statistical analyses performed in Graphpad Prism.

For manuscripts utilizing custom algorithms or software that are central to the research but not yet described in published literature, software must be made available to editors and reviewers. We strongly encourage code deposition in a community repository (e.g. GitHub). See the Nature Portfolio guidelines for submitting code & software for further information.

Data

Policy information about availability of data

All manuscripts must include a data availability statement. This statement should provide the following information, where applicable:

- Accession codes, unique identifiers, or web links for publicly available datasets
- A description of any restrictions on data availability
- For clinical datasets or third party data, please ensure that the statement adheres to our policy

Underlying data are provided as an Excel spreadsheet as Supplemental Data.

Human research participants

Policy information about studies involving human research participants and Sex and Gender in Research.

Reporting on sex and gender	n/a
Population characteristics	n/a
Recruitment	n/a
Ethics oversight	n/a

Note that full information on the approval of the study protocol must also be provided in the manuscript.

Field-specific reporting

Please select the one below that is the best fit for your research. If you are not sure, read the appropriate sections before making your selection.

☒ Life sciences ☐ Behavioural & social sciences ☐ Ecological, evolutionary & environmental sciences

For a reference copy of the document with all sections, see nature.com/documents/nr-reporting-summary-flat.pdf

Life sciences study design

All studies must disclose on these points even when the disclosure is negative.

Sample size	Sample sizes (eg. 10--32 cells per group in this study) were consistent with (or exceeded) similar published datasets (Vyleta and Jonas, 2014; Vyleta and Snyder, 2021).
Data exclusions	To ensure maximum quality of electrophysiology recordings from individual mossy fiber terminals, only recordings with seal resistance greater than 5 giga-ohms were analyzed.
Replication	n/a
Randomization	Mouse litters were randomly chosen to be treated with tamoxifen either during a neonatal period or in adulthood.
Blinding	It was not practical for the experimenter to be blinded to group allocation for these datasets. However, all recorded cells were tested with the same protocols under identical conditions. Furthermore, all analyses were performed without any preconceived expectation of results, and were therefore unbiased.

Reporting for specific materials, systems and methods

We require information from authors about some types of materials, experimental systems and methods used in many studies. Here, indicate whether each material, system or method listed is relevant to your study. If you are not sure if a list item applies to your research, read the appropriate section before selecting a response.

Materials & experimental systems

n/a	Involved in the study	
☒	☐	Antibodies
☒	☐	Eukaryotic cell lines
☒	☐	Palaeontology and archaeology
☐	☒	Animals and other organisms
☒	☐	Clinical data
☒	☐	Dual use research of concern

Methods

n/a	Involved in the study	
☒	☐	ChIP-seq
☒	☐	Flow cytometry
☒	☐	MRI-based neuroimaging

Animals and other research organisms

Policy information about studies involving animals; ARRIVE guidelines recommended for reporting animal research, and Sex and Gender in Research

Laboratory animals	Ascl1CreERT2 mice (Ascl1tm1.1(Cre/ERT2)Jejo; JAX 12882v33) and Ai14 reporter mice (Gt(ROSA)26Sortm14(CAG-tdTomato)Hze; JAX

Laboratory animals	7908)34 were purchased from The Jackson Laboratory, and were crossed to generate offspring that were heterozygous for Ascl1CreERT2 and homozygous for the Cre-dependent tdTomato reporter, as described elsewhere (Yang et al., 2015). Mice maintained on C57Bl/6J background and were used up to 29 weeks of age.
Wild animals	n/a
Reporting on sex	Both sexes are included in this study. No consistent differences were observed so data are pooled. Sex is provided for each recording in the Underlying data spreadsheet in Supplemental Data.
Field-collected samples	n/a
Ethics oversight	All procedures were approved by the Animal Care Committee at the University of British Columbia (UBC) and conducted in accordance with the Canadian Council on Animal Care guidelines regarding humane and ethical treatment of animals.

Note that full information on the approval of the study protocol must also be provided in the manuscript.
